# Mapping QTLs for Salt Tolerance in Rice (*Oryza sativa* L.) by Bulked Segregant Analysis of Recombinant Inbred Lines Using 50K SNP Chip

**DOI:** 10.1371/journal.pone.0153610

**Published:** 2016-04-14

**Authors:** Sushma Tiwari, Krishnamurthy SL, Vinod Kumar, Balwant Singh, AR Rao, Amitha Mithra SV, Vandna Rai, Ashok K. Singh, Nagendra K. Singh

**Affiliations:** 1 National Research Centre on Plant Biotechnology, Pusa Campus, New Delhi, India; 2 Central Soil Salinity Research Institute, Karnal, India; 3 Indian Agricultural Statistics Research Institute, New Delhi, India; 4 Division of Genetics, Indian Agricultural Research Institute, New Delhi, India; Aberystwyth University, UNITED KINGDOM

## Abstract

Soil salinity is a major constraint to rice production in large inland and coastal areas around the world. Modern high yielding rice varieties are particularly sensitive to high salt stress. There are salt tolerant landraces and traditional varieties of rice but with limited information on genomic regions (QTLs) and genes responsible for their tolerance. Here we describe a method for rapid identification of QTLs for reproductive stage salt tolerance in rice using bulked segregant analysis (BSA) of bi-parental recombinant inbred lines (RIL). The number of RILs required for the creation of two bulks with extreme phenotypes was optimized to be thirty each. The parents and bulks were genotyped using a 50K SNP chip to identify genomic regions showing homogeneity for contrasting alleles of polymorphic SNPs in the two bulks. The method was applied to ‘CSR11/MI48’ RILs segregating for reproductive stage salt tolerance. Genotyping of the parents and RIL bulks, made on the basis of salt sensitivity index for grain yield, revealed 6,068 polymorphic SNPs and 21 QTL regions showing homogeneity of contrasting alleles in the two bulks. The method was validated further with ‘CSR27/MI48’ RILs used earlier for mapping salt tolerance QTLs using low-density SSR markers. BSA with 50K SNP chip revealed 5,021 polymorphic loci and 34 QTL regions. This not only confirmed the location of previously mapped QTLs but also identified several new QTLs, and provided a rapid way to scan the whole genome for mapping QTLs for complex agronomic traits in rice.

## Introduction

Rice is the most important cereal crop with maximum contribution to the global food requirements. The world cereal production is about 2500 mt with rice alone contributing 675 mt, accounting for 27 per cent of total [1]. Importance of rice is increasing day by day due to human population growth especially in the Asian countries. Therefore, improving rice productivity is crucial for global food security, economic development and sustainable agriculture. Higher rate of population growth and conversion of highly productive farmlands for industrial and residential purposes have pushed rice cultivation to less productive areas such as saline, sodic, drought and flood prone areas. Globally about 900 mha of farmlands are affected by salinity, which includes both sodic and saline soils [2]. Most traits of agronomic importance in crops, particularly those related to abiotic stress tolerance, are quantitatively inherited. QTLs are genomic regions containing genes controlling these quantitative traits [3]. QTL mapping is one of the most common approaches for the genetic dissection of quantitative traits, which provides the basis for map-based cloning of genes and marker-assisted selection (MAS) in crop breeding. QTL mapping is carried out by genotyping a large number of individuals that are progeny of a bi-parental cross, the process is labor-intensive, time-consuming and costly [4, 5]. BSA provides a shortcut but effective approach to rapidly identify the markers linked to specific genes or QTLs for trait of interest by genotyping only a pair of pooled DNA samples from two sets of individuals with distinct or opposite extreme phenotypes [6, 7]. High-throughput genotyping based on SNP arrays and next generation sequencing (NGS) has evolved very fast during the last decade. Using these technologies, BSA can identify large numbers of markers linked to the target genes or QTLs. One of the first studies to use bulked segregant analysis of RIL population was differential transcriptome analysis to identify candidate genes for salt tolerance using genome wide microarray [8]. Subsequently, a number of studies have been reported on the application of high throughput genotyping for BSA, but they have focused mainly on qualitative traits, e.g. ‘MutMap’ approach or used GBS for genotyping, e.g.‘QTLSeq’ [9–14]. These studies used 5–20 lines with extreme phenotype for creating the bulks and there has been no work on experimental optimization of the number of lines for creating the bulks.

Wolyn *et al*. [15] first proposed an approach named eXtreme Array Mapping (XAM), which combined microarray-based genotyping with BSA and successfully mapped light response QTLs in Arabidopsis, offering an efficient cost effective method of discovering new QTLs. Later studies in yeast (*Saccharomyces cerevisiae*) also demonstrated the effectiveness of microarray-based BSA for QTLs mapping [16, 17]. Ehrenreich *et al*. [18] utilizing NGS as well as microarray-assisted BSA mapped a number of QTLs for 17 chemical resistance traits in yeast (*S*. *cerevisiae*). Magwene *et al*. [19] proposed a statistical framework for QTL mapping based on NGS-assisted BSA. These studies were conducted largely on yeast (*S*. *cerevisiae*), but two recent studies have used NGS-assisted BSA for QTL mapping in rice; Takagi *et al*. [20] mapped several QTLs underlying resistance to rice blast, grain amylase content and germination rate under low temperature, while Yang *et al*. [21] mapped QTLs controlling cold tolerance in rice. A major QTL for panicle erectness in rice was identified by BSA using SSR markers, which was later cloned and characterized in detail [22, 23]. However, this is the first attempt to exploit the BSA approach to identify QTLs for a complex trait in rice using hybridization based high density SNP array. The trait we undertook to map is reproductive stage sodicity tolerance. The detrimental effect of salt stress is reduction of crop yield by alterations in plant metabolism, including reduced water potential, ion imbalances and toxicity. It is often not clear which of the presumed component traits actually contribute to the overall field level salt tolerance of a particular genotype. Integration of results from studies employing diverse approaches on the same mapping population and phenotyping for both, the stress susceptibility index (SSI) and its component traits, is expected to provide the answer but has been rarely attempted [24]. SSI is a better criterion of stress tolerance because it takes into account agronomic performance of a genotype under stress in relation to its yield potential under non-stress condition and the overall stress intensity [25]. Analysis of SSI for economic yield and related traits is important for the crop plants, because often the actual component traits responsible for yield stability under stress are not clearly known. Notable success has been achieved in developing salt-tolerant rice varieties in India through conventional breeding utilizing salt-tolerant landraces, e.g. Damodar, Pokkali, Nona Bokra, and Bhura Ratha, but these varieties have poor yield potential [26]. Salt tolerance is a polygenic trait highly influenced by the environment, which makes it even more difficult to identify the genes and QTLs for use in marker-assisted breeding of the trait into high-yielding varieties. Rice is relatively tolerant to salt stress during germination, active tillering stage and towards maturity, but is highly sensitive to salt during early seedling and reproductive (panicle initiation, anthesis and fertilization) stages [27, 28].

A major QTL for salt tolerance at the seedling stage, namely *Saltol*, was mapped on the short arm of chromosome 1 between RM23 and RM140 (10.7–12.2 Mb), using recombinant inbred lines (RILs) developed from salt tolerant landrace Pokkali and salt sensitive variety IR29 [29]. Thomson *et al*.[30] reported presence of different ‘Pokkali’ alleles in the *Saltol* region between 11.0 Mb and 12.2 Mb and suggested that *Saltol* may involve the *SKC1*gene located at position 11.46 Mb, first identified in Indica landrace Nona Bokra [31]. A study employing F_2:3_ mapping population derived from Nona Bokra and susceptible Japonica variety Koshihikari identified several QTLs for salt tolerance on chromosomes 1, 4, 6, 7 and 9, including major QTLs for shoot K^+^ concentration on chromosome 1 (*qSKC1*) and shoot Na^+^ concentration on chromosome 7 (*qSNC7*) [32]. There are also reports of QTLs for physiological parameters of salt tolerance on chromosomes 3, 4, 6 and 9 [33–35].

QTLs for seedling stage salinity tolerance have been mapped on chromosomes 1, 5, 6 and 7 in earlier studies [36–39], but there is limited information on salt tolerance at reproductive stage (STRS), which is crucial for improving rice productivity under salt stress. Therefore, STRS is an important trait for stable rice production in salt affected areas. Only a limited number of QTLs for STRS have been mapped using low-density SSR markers [40, 41]. Hence, the aim of present study was to rapidly identify additional STRS QTLs and candidate genes for salt sensitivity index (SSI) for grain yield in sodicity tolerant Indica rice variety CSR11 by bulked segregant analysis using high density SNP array.

## Results

### Stress Intensity and Phenotypic Segregation for Salt Tolerance in CSR11/MI48 RILs

The stress intensity (SI) for grain yield under moderate sodic (pH ~9.5) and high sodic (pH ~ 9.9) environments as compared to control micro plots (pH ~7.5) was 0.51 and 0.85, respectively. Homogeneity of error variance across three years of data in 2009, 2010 and 2011 was evaluated by the F test. Combined analyses of variances for genotypes in three seasons were determined by comparing the genotype × season interaction for each trait. Significance levels were also determined for the combined analysis (S1 Table). The CSR11/MI48 RIL population showed significant variability for the nine salt tolerance parameters examined in this study (Table 1).

**Table 1:** Variation for yield and yield contributing traits among 216 RILs derived from CSR11/MI48 over 3 seasons under control (N), moderate sodic (MS) and high sodic (HS) conditions.

	CSR11			MI48			Range in the RILs				
Traits	N	MS	HS	N	MS	HS	N	MS	HS	SD (+/-)	SE (+/-)
DFF	96	102	102	101.9	108.67	112	78.67–113.67	84.00–116.00	88.00–120.50	11.77	1.65
PH	93.33	78	51.8	111.1	78.15	54.91	81.30–174.37	55.12–127.10	31.47–85.23	35.63	7.14
PL	22.6	20	14.5	24.15	19.61	15.86	20.47–32.40	16.37–25.73	10.57–18.75	5.36	1.22
TT	13.83	11.9	9.12	11.15	9.11	6.21	8.80–21.33	6.58–15.63	3.33–11.23	4.39	0.87
PT	12.59	10.7	7.38	10.21	7.85	4.86	7.63–19.70	5.40–14.73	2.38–10.17	4.35	0.9
SW	23.87	21.8	20.3	24.9	20.96	16.55	20.03–30.73	16.65–24.71	11.51–22.38	4.55	0.99
GPP	89.14	81.1	51.9	116.54	66.44	29.32	66.38–167.19	20.68–89.48	6.10–57.02	41.17	10.02
SF	77.41	71.7	59.9	78.46	58.1	40.45	57.79–88.31	33.31–74.05	6.68–63.20	21.32	4.99
GY	12.12	9.42	4.37	16.73	6.2	2.12	6.39–45.76	3.48–15.55	0.29–6.59	11.09	1.78

DFF, Days to 50% flowering; PH, Plant height; PL, Panicle length; TT, Total tillers per plant; PT, Productive tillers per plant; SW, 1000 grain weight; GPP, Grains per panicle; SF, Spikelet fertility; GY, Grain yield per plant; N, Normal; MS, Moderate sodic stress; HS, High sodic stress; RILs, Recombinant inbred lines.

**Fig 1:**
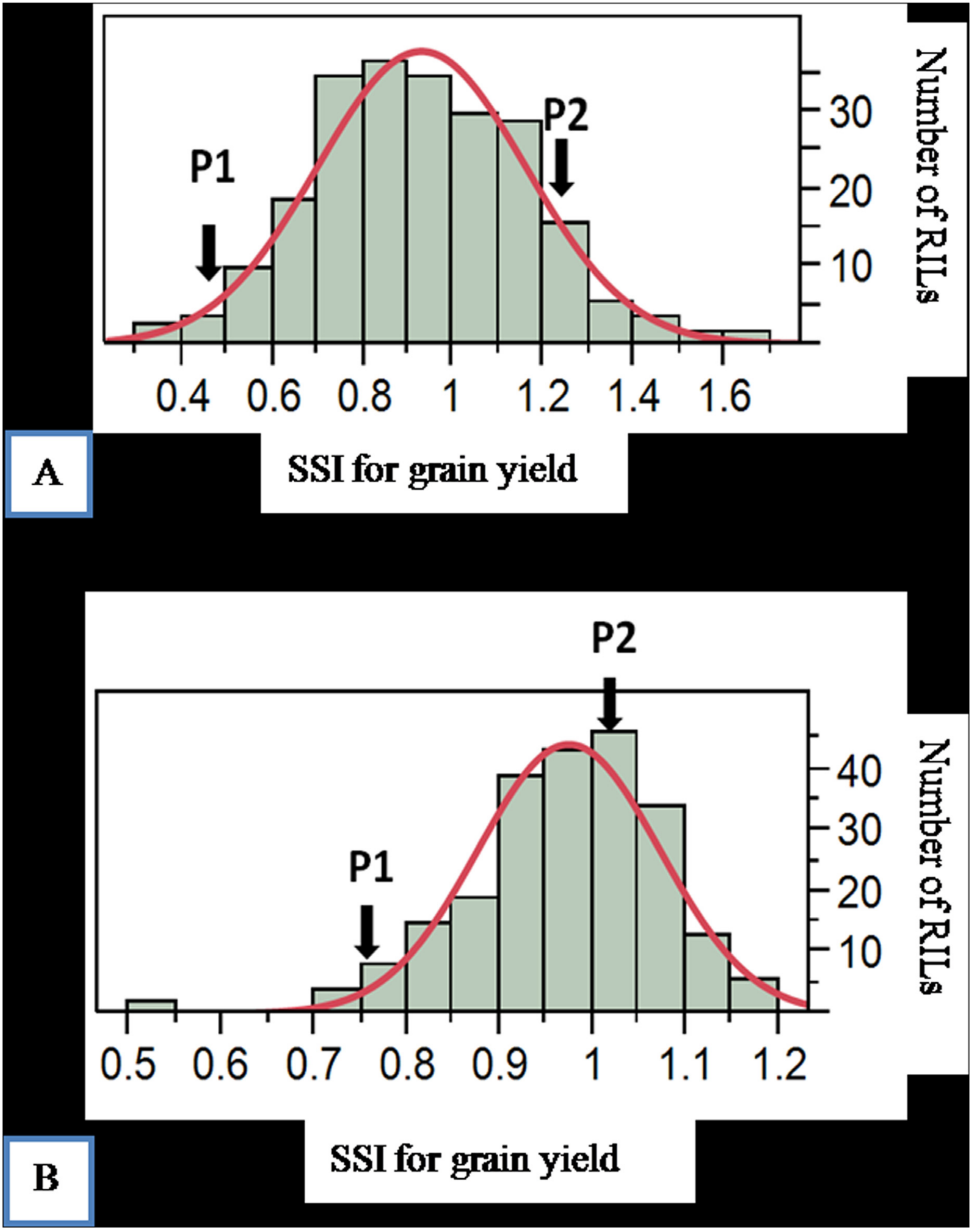
Frequency distribution of SSI for grain yield among CSR11/MI48 RILs under. (A) Moderate sodicity and (B) High sodicity.

All the parameters showed transgressive segregation and near normal distribution, suggesting involvement of multiple genes with quantitative inheritance (S1 Fig). Analysis of variance showed that variance for yield and yield contributing traits in the RILs was significant (P < 0.01). The RIL population showed mean grain yield per plant of 15.04, 7.39 and 2.21 under normal, moderate and high sodicity, respectively (Table 1). The tolerant parent CSR11 expressed mean yield of 12.12, 9.42 and 4.37; whereas the sensitive parent MI48 expressed mean yield of 16.73, 6.20 and 2.12 under normal, moderate and high sodicity, respectively. The tolerant parent CSR11 showed significantly less grain yield reduction of 22% than the susceptible parent MI 48 showing 63% yield reduction under moderate sodicity. The tolerant parent CSR11 showed lower SSI for yield under moderate (0.44) and high sodicity (0.75) as compared to the sensitive parent MI48 showing SSI of 1.24 under moderate and 1.02 under high sodicity across three seasons (Table 2, Fig 1, S1 Fig).

**Table 2:** Variation for salt stress susceptibility index for different traits among 216 RILs derived from CSR11/MI48 over 3 seasons.

Traits	CSR11			MI48			Range RILs		
	MS	HS	Mean ± SE	MS	HS	Mean ± SE	MS	HS	SE
SSI DFF	1.46	0.89	1.17±0.02	1.53	1.52	1.52±0.02	- 0.49 to—2.57	0.07–2.75	0.09
SSIPH	0.70	0.93	0.81±0.12	1.26	1.05	1.16±0.16	0.44–1.81	0.67–1.46	0.06
SSIPL	0.73	0.97	0.85±0.13	1.20	0.93	1.06±0.13	0.30–1.90	0.50–1.53	0.04
SSITT	0.88	0.92	0.9±0.14	1.17	1.19	1.10±0.20	-0.17 to—3.04	0.51–2.28	0.05
SSIPT	0.65	0.77	0.71±0.19	0.97	0.97	0.97±0.27	-0.29 to—1.97	0.38–1.63	0.05
SSISW	0.60	0.51	0.56±0.14	1.08	1.14	1.11±0.12	0.25–1.71	0.44–1.70	0.09
SSIGGP	0.21	0.61	0.41±0.13	1.00	1.09	1.04±0.15	0.29–1.80	0.63–1.37	0.11
SSISF	0.36	0.47	0.41±0.11	1.25	1.01	1.13±0.15	0.02–2.66	0.33–1.90	0.11
SSIGY	0.44	0.75	0.59±0.25	1.24	1.02	1.13±0.24	0.32–1.62	0.71–1.16	0.09

SSI, Stress susceptible index; DFF, Days to 50% flowering; PH, Plant height; PL, Panicle length; TT, Total tillers per plant; PT, Productive tillers per plant; SW, 1000 grain weight; GPP, Grains per panicle; SF, Spikelet fertility; GY, Grain yield per plant; N, Normal; MS, Moderate sodic stress; HS, High sodic stress; RILs, Recombinant inbred lines

The top and bottom 30 RILs with extreme phenotypes were selected based on consistent SSI for grain yield across the two levels of stress, moderate and high sodicity in three seasons (2009, 2010 and 2011, S2 Table). The SSI for grain yield ranged from 0.54 (RIL106) to 0.9 (RIL9) for top 30 RILs whereas, for bottom 30 RILs it ranged from 1.03 (RIL 20) to 1.38 (RIL 131) (S2 Fig). Analysis of correlation coefficients revealed the nature and magnitude of association of yield and yield component traits among themselves and with SSI under different sodicity regimes (Table 3). Grain yield per plant showed significant positive association with plant height, panicle length, total tillers per plant, productive tillers per plant, 1000 grain weight, grains per panicle and spikelet fertility (Table 3). As expected the number of productive tillers/plant was positively correlated with total number of tillers/plant in all salt environments.

**Table 3:** Correlation coefficients of yield and yield component traits with SSI for grain yield under normal, moderate and high sodicity regimes in CSR11/MI48 RILs.

**Traits**	**Stresses**	**DFF**	**PH**	**PL**	**TT**	**PT**	**SW**	**GPP**	**SF**	**GY**
PH	N	0.10*								
	MS	0.12*								
	HS	0.12*								
PL	N	-0.01	0.71**							
	MS	0.04	0.81**							
	HS	0.15*	0.85**							
TT	N	0.08	0.21**	0.19**						
	MS	0.19**	0.19**	0.21**						
	HS	0.12*	0.29**	0.31**						
PT	N	0.04	0.24**	0.20**	0.95**					
	MS	0.16**	0.25**	0.26**	0.90**					
	HS	0.08	0.34**	0.36**	0.85**					
SW	N	0.05	0.11*	0.12*	0.09	0.17*				
	MS	0.08	0.45**	0.48**	0.25**	0.40**				
	HS	-0.01	0.43**	0.47**	0.27**	0.43**				
GPP	N	0.27**	0.37**	0.38**	0.21**	0.25**	0.15*			
	MS	0.09	0.46**	0.48**	0.18**	0.29**	0.39**			
	HS	0.01	0.53**	0.52**	0.17*	0.23**	0.39**			
SF	N	0.09	0.24**	0.21**	0.38**	0.49**	0.21**	0.49**		
	MS	0.05	0.22**	0.22**	0.31**	0.46**	0.45**	0.69**		
	HS	-0.12*	0.43**	0.40**	0.14*	0.22**	0.45**	0.80**		
GY	N	0.20**	0.45**	0.46**	0.61**	0.64**	0.35**	0.58**	0.54**	
	MS	0.12*	0.43**	0.45**	0.51**	0.62**	0.55**	0.58**	0.60**	
	HS	0.01	0.59**	0.57**	0.54**	0.65**	0.62**	0.49**	0.44**	
SSI GY	MS	0.21**	-0.18**	-0.21**	0.12*	0.07	-0.15*	-0.21**	-0.10*	-0.26**
	HS	0.12*	-0.28**	-0.25**	-0.30**	-0.34**	-0.21**	-0.28**	-0.15*	-0.58**

PH, Plant height; PL, Panicle length; TT, Total tillers per plant; PT, Productive tillers per plant; SW, 1000 grain weight; GPP, Grains per panicle; SF, Spikelet fertility; SSI, Stress susceptible index; GY, Grain yield per plant; N, Normal; MS, Moderate sodic stress; HS, High sodic stress; RILs, Recombinant inbred lines;

* and **, significant at 0.1 and 0.5 level respectively

The spikelet fertility showed significant positive correlation with all other traits except days to 50% flowering in all salt stress environments. Similarly, panicle length showed significant positive correlation with plant height, total tillers in all salt stress environments and controls. Correlation coefficients of morphological traits with SSI for grain yield under moderate and high sodicity revealed that SSI for grain yield had significant negative correlation with plant height (-0.18 and -0.28), panicle length (-0.21and -0.25) and grain per panicle (-0.21 and -0.28), respectively.

### Optimization of Pool Size for Bulked Segregant Analysis

**Fig 2:**
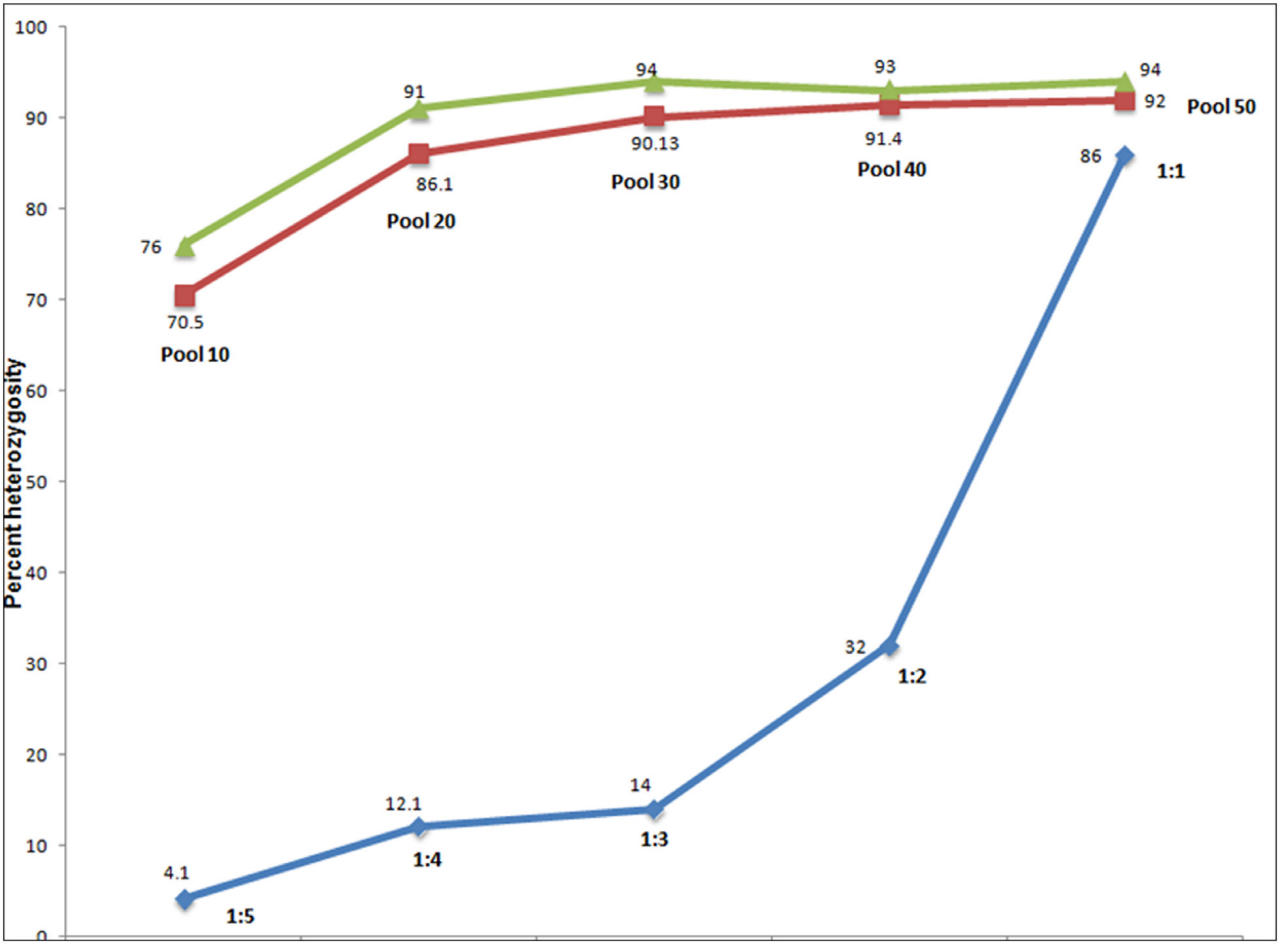
Analysis of heterogeneous loci in different RIL pool sizes and mixture of parental DNA samples using 50K SNP chip. Genomic DNA from 10 to 50 individual RILs of CSR11/MI48 mapping population were pooled in equal amounts, with higher pools including all the RILs of lower pools for the analysis of allele heterogeneity (Red line). Computational expectations on Bootstrap analysis of the pools of 10 to 50 lines, showing successive increase in heterogeneity up to 94% (Green line). Observed heterogeneity with mixing of genomic DNA from the two parental lines in the proportions of 1:5, 1:4, 1:3, 1:2 and 1:1 (Blue line).

Array based SNP genotyping of pools of 10, 20, 30, 40 and 50 RILs was done to identify the optimum pool sizes for maximum heterogeneity (heterozygote calls) of alleles in the pool, so that the bulked tolerant and bulked sensitive pools did not differ for alleles other than those in the associated QTL regions. Out of 50,051 SNPs on the chip array, 41,327 were monomorphic in all the pools and 2,903 had missing calls in one or more pools. Hence, we analyzed the remaining 5,821 SNPs that showed heterogeneity in any of the five pools. As expected, pool of 10 RILs showed the minimum number of 4,115 (70%) heterogeneous SNPs, with successive increase in heterogeneity in higher pools of 20 (86.1%), 30 (90.13%), 40 (91.4%) and 50 (92%) RILs (Fig 2, S3 Table). Thus, our empirical study showed that a pool of 10 RILs exhibiting only 70% heterogeneity was not suitable for BSA, as it would give about 30% false positive associations. The optimum pool size was of 30 RILs resulting in >90% of the loci with heterogeneous SNPs in the pool to minimize the number of false associations. Pools with more than 30 RILs did not offer further significant increase in heterogeneity. The SNP heterogeneity increased with the increasing pool size, but maximum gain was between pool size of 10 to 30 RILs, wherein it increased from 70% to 90.2%, after which there was negligible increment in heterogeneity; less than two percent after adding another 20 RILs to the pool. Since sampling for different pool size was done in such a way that the lower pool was a subset of the higher pool, we carried out bootstrapping analysis to draw inference on the population rather than just the representative samples. Bootstrapping results also indicated that only 76% heterogeneity was predicted with the pool size of 10, whereas 94% heterogeneity was predicted with pool size of 30, after which no further significant increase was predicted, which was close to the experimental results (Fig 2). We could not get 100% heterogeneity in any of the pools examined either experimentally or computationally by bootstrapping. We also tested the sensitivity of Affymetrix array in detecting minor alleles in the pool of RILs by genotyping physical mixtures of DNA from the two pure parental lines in different proportions (1:1, 1:2, 1:3, 1:4, 1:5). We intended to find the level at which a minor allele in the pool ceases to be resolved by the array. Out of 50, 051 SNPs on chip array, we analyzed 4, 245 loci, which were polymorphic between the two pure DNA samples of CSR11 and MI48. Only 4.1% of the polymorphic loci showed heterozygote calls at the lowest minor allele frequency of 20% with 1:5 ratio of the two DNA samples, with successive increase in heterozygosity with 1:4 (12.1%), 1:3 (14%), 1:2 (32%) and 1:1 (86%) (Fig 2). Surprisingly, even with supposedly equal frequency of the two alleles as in 1:1 mixture; only 86% of the loci were called heterozygote, leaving an error margin of 14% which was even higher than 10% homozygosity of alleles observed in the pool of 30 extreme RIL. This would lead to false associations and an over estimation of QTLs. However, in our BSA approach both bulked-tolerant and bulked-sensitive pools must show homogeneity for contrasting alleles for a QTL call, which would drastically decrease the probability of false QTL discovery to 0.10^2^ = 0.01).

### QTLs Identified in CSR11/MI48 RIL Population Using BSA Approach

**Fig 3:**
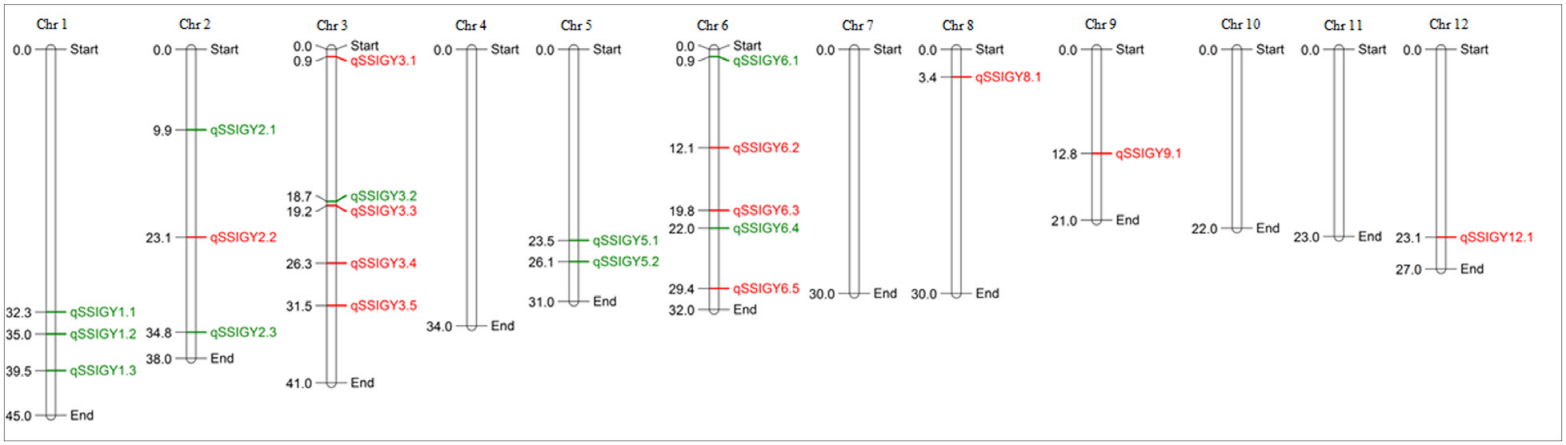
Physical map positions of QTLs identified by BSA of CSR11/MI48 RIL population using 50K SNP chip. QTLs shown in green color have salt tolerant allele coming from the tolerant parent CSR11 and those in red color have tolerant allele contributed by the sensitive parent MI48.

The BSA method using high density SNP genotyping was applied to CSR11/MI48 RILs segregating for sodicity tolerance and several new QTLs were identified for SSI for grain yield. After optimization of the RIL pool size, SNP genotyping of CSR11, MI48 and the bulked-tolerant (BT) and bulked-susceptible (BS) pools of 30 RILs each were done using 50K rice SNP chip. Out of 50,051 SNPs on the chip we have analyzed 6,068 SNPs that were polymorphic between BT and BS pools (S4 Table). Interestingly, the sensitive parent MI48 analyzed here differed significantly from the original MI48 used in the cross to develop the mapping population, as the parental lines were monomorphic but the RILs were polymorphic at 1,692 loci. In such cases the allele call of CSR11, the properly maintained parent, was considered as true call and the alternate allele was assigned to MI48. We could have ignored these loci from the analysis but that would have resulted in missing out on some important QTLs. Thus, all 6,068 polymorphic loci were categorized into five groups in S4 Table; group 1 with both BT and BS heterogeneous (4809 loci, white rows); group 2 with tolerant allele for the QTL was contributed by CSR11 having one heterozygous locus (129 loci, green rows); group 3, where both the loci were homogeneous and tolerant allele was contributed by tolerant parent CSR11 (11 loci, dark green rows); group 4 where tolerant allele was contributed by the sensitive parent MI48 and one locus was heterozygous (1108, red rows) and group 5 where both the loci were homogeneous and tolerant allele was contributed by MI48 (11 loci, dark brown rows). As shown in our pool optimization study, about 10 percent of the loci showed homozygote calls even with the pool of 30 RILs, which would lead to false discoveries of QTLs. Clearly, there was an issue with the sensitivity of the Affymetrix chip for the detection of minor alleles in the pooled DNA samples. To minimize the false discovery, we assigned QTL status to a SNP or a string of SNP loci only when both the pools were homogeneous for contrasting alleles (group 3 and group 5), which will drastically reduce the false QTL calls. This way, we identified total 21 QTLs for SSI for grain yield on rice chromosomes 1, 2, 3, 5, 6, 8, 9 and 12 in the CSR11/MI48 RILs (Fig 3, S5 Table).

Three QTLs were identified on chromosome 1 at positions 32.3, 35.0 and 39.5 Mb with tolerant alleles contributed by CSR11. A homogeneous SNP was identified in single copy rice gene Os01g55530 at 32.3 Mb, which is reported to be involved in metabolism, signal transduction, and response to stress. Three QTLs were identified on chromosome 2, namely *qSSIGY2*.*1*, *qSSIGY2*.*2* and *qSSIGY2*.*3* at positions 9.9, 23.1 and 34.8 Mb. Source of tolerance for QTLs *qSSIGY2*.*1* and *qSSIGY2*.*3* was CSR11 and at *qSSIGY2*.*2* tolerant allele was coming from MI48. The QTL *qSSIGY2*.*1*, was marked by a homogeneous SNP in single copy rice gene Os02g17190 (MYB family transcription factor) know to be associated with stress tolerance. Five QTLs were mapped on rice chromosome 3 and tolerant alleles in four of these were from sensitive parent MI48 and one from the tolerant parent CSR11. QTL *qSSIGY3*.*5* was marked by SNP in the gene Os03g55490 having kinase activity. Two QTLs were mapped on chromosome 5 at 23.5 and 26.1 Mb positions with tolerant allele contributed by CSR11. At 26.1 Mb, homogeneous SNPs were present in two consecutive genes (Os05g45040, Os05g45050), but it was considered one QTL because of their adjacent location. Five QTLs were identified on rice chromosome 6, out of which three had tolerant allele coming from CSR11 and remaining three from MI48. One QTL was mapped on rice chromosome 8, marked with SNP in gene Os08g06110, which is MYB transcription factor. One QTL was identified on rice chromosome 9 and source of tolerant allele for SSIGY was MI48. In the gene Os09g02790 that codes for a zinc binding protein. Another QTL was identified on rice chromosome 12 at position 23.1 Mb with tolerant allele coming from sensitive parent MI48. We found that out of 21 QTLs reported here, five were in the regions reported earlier in the CSR27/MI48 F_2_/F_3_ and RILs with one common parent [8, 42], the remaining 15 were novel QTLs for salt tolerance in the CSR11/MI48 RILs, with CSR11 having a different mechanism of sodicity tolerance as compared to CSR27.

### Validation of BSA Approach with CSR27/MI48 RIL Population

**Fig 4:**
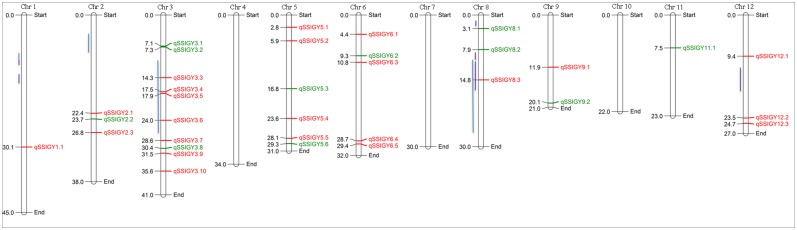
QTL positions identified in CSR27/MI48 population by BSA using 50k SNP chip. Physical map position of QTLs with green color showing tolerant allele coming from tolerant parent CSR27 (11 loci), red color showing tolerant allele coming from sensitive parent MI48 (23 loci). Blue and violet bars represent earlier identified QTLs by Ammar *et al*. [42] and Pandit *et al*. [8], respectively.

We did bulked segregant analysis of BT and BS pools of 30 RILs each along with the parental lines CSR27 and MI48 using 50K SNP chip to validate the QTLs reported earlier in this population [8, 42]. Out of 50,051 SNPs present on the chip, we analyzed 5,021 SNP loci, which were polymorphic between BT and BS, and categorized them into five groups as described above; group 1, where both BT and BS were heterozygous (3999 loci, white rows); group 2, with tolerant allele for the QTL was contributed by CSR27 having one heterozygous locus (691 loci, green rows); group 3, where both the loci were homogeneous and tolerant allele was contributed by tolerant parent CSR27 (11 loci, dark green rows); group 4, where tolerant allele was contributed by the sensitive parent MI48 and one locus was heterozygous (294, red rows) and group 5, where both the loci were homogeneous and tolerant allele was contributed by MI48 (23 loci, dark brown rows) (S6 Table). For QTL calling we considered only those loci, which were homogeneous in both the pools. This way we identified total 34 QTLs on rice chromosomes 1, 2, 3, 5, 6, 8, 9, 11 and 12 (Fig 4). BSA approach not only validated most of the earlier reported QTLs but also identified several new QTLs because of the high resolution of SNP markers. In this mapping population, tolerant allele of SSIGY QTLs was from tolerant parent CSR27 at 11 QTL positions (group 3), whereas tolerant allele was coming from sensitive parent MI48, at 23 QTL positions (group 5, S7 Table).

Earlier, Ammar *et al*. [42] using CSR27/MI48 F_2_/F_3_ populations have mapped 25 major QTLs on chromosomes 1, 2, 3 and 8 and Pandit *et al*. [8] have mapped QTLs on chromosomes 1, 8 and 12 using RIL populations of the same cross. On chromosome 1, Ammar *et al*. [42] has reported five QTLs between 9.86–10.07 Mb position and Pandit *et al*. [8] has reported four QTL regions between 8.32–10.8Mb and 11.02–15.87 Mb positions, here we found only one QTL on chromosome 1 at 30.1 Mb position considering the strict criteria of allelic homogeneity in both the bulks. However, two of the previously mapped QTLs located in the regions showing homogeneity of alleles in at least one bulk (S6 Table).

Ammar *et al*. [42] had reported QTL, *qNa/KSV2*.*1/qNa/KSV2*.*2*, *qNa/KSV2*.*3*, *qClLR-2*.*1*, *qClSV2*.*2* at 4.4–8.98 Mb on chromosome 2. This QTL was not validated in the present study with the strict criteria of homogeneity in both the pools but at two positions (4.2–4.3 Mb, 8.4–8.5 Mb) homogeneity of alleles in at least one bulk was present (S6 Table). We found three new QTLs between 22.4 and 26.8 Mb position two of them *qSSIGY2*.*1* and *qSSIGY2*.*3* were contributed by MI48 and at one QTL position tolerant allele was coming from CSR27. Ammar *et al*. [42] reported most important QTL on chromosome 3, which influenced nine of the seventeen salt tolerance parameters studied between markers RM563-RM186 (11.07 to 28.8 Mb). Here we have found 5 QTLs for SSIGY at 14.3, 17.5, 17.9, 24 and 28.6 Mb regions, source of tolerant allele was MI48 in all of them.

Ammar *et al*. [42] has reported QTL for six parameters between marker intervals RM3395-RM281 (10.2–27.89 Mb) on chromosome 8. We found one QTL *qSSIGY8*.*3* at 14.3 Mb position, where tolerant allele for QTL region was coming from MI48. Pandit *et al*. [8] reported 4 QTLs on chromosome 8, *qNaSH-8*.*1* (1.208–2.8 Mb), *qClLV-8*.*1a* (9.2–10.29 Mb), *qClLV-8*.*1b* (10.29–19 Mb), and *qSSISFH-8*.*1* (9.2–10.29 Mb). Here we found three QTL regions at 3.1, 7.9 and 14.8 Mb positions which are nearby the *qNaSH-8*.*1*, *qClLV8*.*1b and qSSISFH8*.*1* QTLs. They reported most significant QTL for SSI for spikelet fertility at high salt concentration *qSSISFH*-*8*.*1*on chromosome 8 in the marker interval HvSSR08-25-RM3395 (9.2–10.29 Mb). This QTL was co-located with *qClLV*-*8*.*1a* for Cl^-^ content in the leaves at vegetative stage. We found two QTLs, *qSSIGY8*.*2* (7.9 Mb), with tolerant allele coming from CSR27 and *qSSIGY8*.*3* (14.8 Mb), with tolerant allele coming from MI48. On chromosome 12, Pandit *et al*. [8] reported a QTL *qNaSV-12*.*1* between 12.11–17.53 Mb. In our study we found two QTL regions at 9.4 and 23.5 Mb position, at 11.2–14.8 Mb position one QTL region was showing one homogeneous and one heterogeneous locus.

In our study out of 34 QTLs of CSR27/MI48 population five QTLs were reported earlier in the and found 29 novel QTL regions on rice chromosomes 1, 2, 3, 5, 6, 9, 11 and 12 due to dense SNP map of polymorphic locus covering all regions of the genome. Earlier highest 41 QTLs have been reported by Ghomi *et al*. [43] on all the 12 rice chromosomes for salinity tolerance at seedling stage in rice. There are several reports on QTL mapping for salt stress by SSR genotyping on whole population in rice [32–37, 40–44] but no one has done QTL mapping by BSA approach for salt stress in rice.

## Discussion

### Stress Intensity and Segregation for Sodicity Tolerance in the RILs

Analysis of variance revealed highly significant variation among the RILs for all the nine traits under the three stress environments, suggesting the presence of sufficient genetic variation and ample scope for the improvement of rice for reproductive stage sodicity tolerance. The stress intensity of phenotyping for grain yield indicates the moderate and high degree of sodic stresses. The degree of high sodic stress was almost two times more than that of moderate sodicity. Hence, the population expressed 70 per cent grain yield reduction under high stress over moderate sodic stress. Fernandez [45] has shown that association between stress yield (Y_s_) and non-stress yield (Y_p_) was 0.46 for the moderate stress and 0.22 for severe stress conditions, which was almost similar to the present study for moderate sodicity (0.63) and high sodicity (0.17) stress.

Grain yield is a complex quantitative trait, greatly affected by environment. Hence, selection of superior genotypes based on yield per se is not effective. The association of plant characters and stress indices with yield thus, assumes special importance in formulation of selection criteria for yield. Stress susceptibility index (SSI) has been proposed for identifying genotypes with superior performance under stress as well as non-stress environments [25]. It is worthwhile to knowing the association between of stress susceptible index and various agro-morphological traits through simultaneous evaluation under normal and sodicity conditions to draw parallels and differences across important traits for selection in the target environments. The extreme tolerant and sensitive RILs were selected based on their SSI for grain yield, which was a direct measure of salt tolerance and therefore would provide independent line of evidence to those based on salt tolerance parameters. These associations could be useful in identifying salt tolerant and high yielding genotypes [46]. SSI for economic yield has also been used for evaluation of hexaploid triticale × bread wheat introgression lines for tolerance to low phosphorus and water stresses and was found important for selection of efficient genotypes [47]. Porch *et al*. [48] also reported that several genotypes of common beans were superior for heat tolerance based on the stress index and on the consistency of their reactions across environments. Selection of genotypes under normal, moderate and high sodic conditions should be entirely different as indicated by differentials in magnitude of correlation in different salt stress and non-stress conditions in rice [49, 50]. Ali *et al*. [51] have indicated that yield traits in rice such as grains per panicle, spikelet fertility, plant height, productive tillers and flowering duration were good indicators for selecting salt tolerant genotypes in comparison to non-stress. As grain yield is a complex trait with large environmental influence, this along with salt tolerance, also a complex trait governed by multiple genes, makes improvement of yield under salt stress even a tougher challenge.

### QTL Identification by BSA with High-Density SNP Chip

The present method of QTL mapping by BSA using high-density SNP chip provides a rapid and efficient means for the identification of molecular markers linked to complex agronomic traits. BSA method was first applied to identify markers linked to specific genes in F_2_ population using RFLP and RAPD markers [5, 6]. Genotyping with traditional gel based molecular markers is laborious and time-consuming. In recent years NGS-assisted BSA have been utilized to genotype mapping populations [8, 16, 52–55]. This is especially useful when there is limited SSR polymorphism available in the region of interest. Here we used SNP chip array genotyping to ensure rapid data generation and analysis in a simple and cost effective manner. The 50K SNP chip [56], incorporates 48,211 SC gene based non-redundant high quality SNPs probes. It has a high sample success rate (99.4%), SNP call rate (99.9%) and assay reproducibility (99.9%). In BSA an important question is how many individual samples should be pooled for the creation of bulks. Michelmore *et al*. [5] say that frequency of false positive will increase in smaller size bulks and if many loci are screened, probability of getting linked locus will increase. Giovannoni *et al*. [6] took DNA pools containing 7 to 14 F_2_ individuals and concluded that pooling larger numbers of individuals increases the probability that the two pools will not differ for alleles other than those linked with the trait. They have concluded that if the marker interval is larger, then the pooling size should be smaller. As the marker interval increases to 10 cM, fewer than 10 individuals can be pooled to maintain the same probability of double crossovers. Thus the conclusion is to use more number of individuals to create the bulks and simultaneously decrease the size of marker intervals examined. In the present study with high density genotyping we found empirically that a pool of 10 RILs was not enough (only 70% heterogeneity) and at least 30 RILs should be pooled to get heterogeneity for more than 90 percent of the loci in order to reduce the number of false positives. As expected the heterogeneity increased with the increasing pool size, but the maximum gain was between pool 10 to 30 where it increased from 70% to 90.2%, after this there was very little increment of heterogeneity; less than 2 percent by adding another 20 RILs to the pool. Hence, a pool of 30 RILs was considered optimal. Magwene *et al*. [19] based on simulation studies suggested using bulk sizes as large as 15–20% of the population to increase power of detecting causal QTLs, even though this implies weaker selection and less extreme allele frequency differences. In our study also, the bulk size has worked out to 14% (30/216) of the population size, though the size was determined empirically. On the other hand, if one wants to do away with weaker selection, the base population size can be increased. We describe here the first report of experimentally determining the optimum pool size for BSA using high density SNP chip.

High density array chip however has the limitation of not being able to detect all the heterogeneous loci as we have observed in 1:1 ratio of two DNA samples which could detect only 86% loci as heterozygous, which is far below the expected 100%. Thus the high-density chip array has some inherent limitations of not being able to detect the allelic heterogeneity in the pool accurately. This is apparently because the Affymetrix allele-calling algorithm has been optimized for inbred individuals (homozygous) calls and this is presently being improved for the true heterozygote calls for pooled population samples with minor alleles. In our study of QTL mapping by BSA, this would lead to identification of false positive associations as QTLs by 1%. Earlier Takagi *et al* [54] described a method named ‘QTL seq’, where DNA sequence reads were aligned to reference sequence of either of the parents, and SNP-index plots of H-bulk and L-bulk were generated. Genomic regions displaying contrasting patterns of SNP-index between the two bulks defined the QTL positions. Following this, one major concern in our study was whether to consider the homogenous SNPs alone as QTLs or look at the haplotypes in the vicinity of the linked SNP since this is a high density assay. For this we devised a scoring method where genotype calls of flanking markers of all the QTL-linked SNPs were considered except where the SNPs themselves were at ends of the chromosome. There was only one such exception in one of the mapping populations (a SNP in chromosome 9 in the mapping population derived from CSR11/MI48). The flanking markers were assigned a score of one when they were of parental type; heterozygous recombinants received a score of 0.5 whereas homozygous recombinants were given a score of zero. The results revealed that not even a single linked SNP obtained the maximum possible score of four, wherein the flanking markers in both the bulks and in both the adjacent regions would have been of respective parental types (S8 Table). It is worthwhile to mention here that the average size of a single QTL region considered for the above analysis was 575 kb in one population and 604 kb in another. This translates to an average genetic distance of 2.3 cM (@250 kb per cM). Despite such narrow genetic distances, we did observe recombination events routinely since we were handling advanced RIL populations. Based on the above analysis and observations we decided to concentrate only on the homogeneous bulks rather than the haplotypes of nearby flanking SNPs.

At reproductive stage there are very few reports on QTL mapping for salt stress. Recently Hossain *et al*. [41] has reported several QTLs on chromosome 1, 7, 8 and 12 at reproductive stage for salt tolerance in rice. They have reported 11 QTLs at 31.06 and 32.67Mb position on chromosome1. In the mapping population CSR11/MI48 three QTL regions were present between 32.3 to 39.5 Mb position and in other mapping population CSR27/MI48 one QTL region was present at 30.1 Mb position. Out of 21 QTLs found in CSR11/MI48 RILs 5 QTLs were similar to QTLs reported in our study in RIL population CSR27/MI48 by BSA.

For validation of 50K BSA approach it was compared with the SSR based QTL mapping described earlier by Ammar *et al*. [42] and Pandit *et al*. [8] in the same bi-parental population of CSR27/MI48. We found 5 common QTLs reported earlier by SSR genotyping of whole mapping population and identified additional 29 QTLs in CSR27/MI48 mapping population due to dense SNP map of 5,021 polymorphic loci. The QTL mapping has twin problems of underestimation and overestimation of QTL. The present approach with high marker density ensures that rarely a QTL will be missed. However, a large pool size created from a small RIL population will also lead to missing QTLs due to heterogeneity of RILs in the pool for lack of sufficient number of RILs in the base population that have accumulated all the QTLs. A mapping population of RIL would segregate into 1:1 ratio for SNPs. If there are five unlinked genes governing a complex trait, to identify a single plant having all the favorable allele, a population of 95 is required. To pool 10 such individuals, which is common practice in BSA based studies, one needs at least 506 lines [57]. Hence, a larger RIL population size is better to select actual extreme phenotype having all the favorable allele for BSA. To minimize the overestimation of QTL regions we have considered only those loci which were homogeneous in both tolerant and sensitive pools, as both the pools could not became homogeneous for the same loci just by chance.

A major disadvantage of QTL mapping by BSA is that we do not have an estimation of the proportion of variation explained by the QTL. For this at least we need to genotype sufficient number of individual RILs so that effect of different combination of genes can be examined. This individual RIL genotyping could be restricted to only those potential QTLs (SNPs) identified by BSA so that the entire procedure remains cost effective. The method of QTL mapping we have reported here is a fast method to make physical map of any particular trait using a reasonably high density array, which is cost effective and time saving.

## Materials and Methods

### Plant Material

A mapping population of 216 recombinant inbred lines (RILs) was developed from a cross between rice varieties CSR11 and MI48 using single seed descent method. CSR11 is a salt-tolerant, Indica rice variety bred at Central Soil Salinity Research Institute (CSSRI), Karnal, India. It is a Na^+^ excluder and has tolerance to sodic (pH 9.6–9.9) soils [26]. In contrast, MI48 is a salt-sensitive Indica variety. A second mapping population of 216 RILs from CSR27/MI48,used earlier for mapping salt tolerance QTLs with SSR and SNP markers [8], was employed for validation of the QTLs by BSA using 50K SNP array based SNP genotyping.

### Phenotyping for Salt Stress Susceptibility Index and Preparation of Bulks

Phenotyping of CSR11/MI48 RILs for salt tolerance parameters was commenced on the stabilized F_10_ plants of 216 RILs along with two parents. The phenotyping was performed in 2009 (F_10_ plants), 2010 (F_11_ plants) and 2011 (F_12_ plants) under precisely controlled micro plots having two stress levels, moderate sodicity (pH ~ 9.5) and high sodicity (pH ~ 9.9), and non-stress (pH ~ 7.5) control plots at CSSRI, Karnal. Thirty day-old seedlings were transplanted in concrete microplots in simple lattice design for evaluation under normal, moderate and high sodicity stresses. At each stress level, genotypes were replicated twice with row×plant spacing of 20×15 cm. This micro plot facility has been created using brick mortar concrete materials having dimension of 6 m (length), 3 m (width) and 1 m (depth) under rainout shelter. The lysimeters were filled with soil which is uniform throughout the depth. The desired levels of moderate sodicity (pH ~ 9.5) and high sodicity (pH ~ 9.9) simulating the natural field conditions but without the field soil heterogeneity were created by adding required amount of NaHCO_3_ in the soil. The CSR27/MI48 RIL population was phenotyped for salt stress susceptibility index and other salt tolerance parameters as described earlier [8]. Data on five plants from each RIL plot were recorded on grain yield and other component traits namely, days to 50% flowering (DFF), plant height (PH), panicle length (PL), total tiller number (TT), productive tiller number (PT), 1000 seed weight (SW), grains per panicle (GPP), spikelet fertility (SF), and grain yield (GY) during kharif 2009, 2010 and 2011. Thirty each of most tolerant and most sensitive RILs of CSR11/MI48 mapping population were identified based on SSI for GY to create the two bulks. Extreme top and bottom RILs were selected based on the consistence performance of SSI for grain yield for three years (2009, 2010 and 2011) under moderate and high sodicity. For the optimization of number of RILs to be pooled for BSA, bulks of 10, 20, 30, 40 and 50 random RILs were created with each larger bulk including all the lines of the smaller bulks and analyzed with 50K SNP chip for allelic heterogeneity.

### Statistical Analysis

Homogeneity of error variance across the three seasons was tested by the F test [58] and combined analyses of variance for genotypes were performed. Significance levels were determined for the combined analysis [59]. Stress susceptibility index (SSI) was calculated according to Fischer and Maurer [23] and correlation coefficients among traits were computed as per the formulae suggested by Miller *et al*.[60] using SAS 9.3 statistical package.

### Plant DNA Extraction, SNP Genotyping and Optimization of Pool Size

For optimization of RIL pool size for BSA, genomic DNA was isolated from 50 random RILs of the CSR11/MI48 mapping population using CTAB method with minor modifications [61]. Purified DNA (20ng/ul) was combined in equal amounts to prepare different pool size of RIL numbers of 1–10, 1–20, 1–30, 1–40 and 1–50. A custom designed 50K SNP chip based on single copy genes, covering all the 12 rice chromosomes with an average interval of less than 1 kb between adjacent SNP markers was used for high throughput genotyping [56]. Affymetrix Axiom^®^ 2.0 Assay Manual Target Prep Protocol was followed for DNA amplification, fragmentation, chip hybridization, single-base extension through DNA ligation and signal amplification. After Staining and scanning on the GeneTitan^®^ Multi-Channel Instrument CEL file was extracted using the Affymetrix Genotyping Console^™^ v4.1 software package. SNP annotation library was used as standard reference set and development quality check was calculated. Threshold DQC (>0.85) and higher SNP call rate (>95%) was used for further analysis. To see the technical difficulties and sensitivity of array different proportion of two pure DNA samples of CSR11 and MI48 were used in ratio of 1:1, 1:2, 1:3, 1:4 and 1:5.

### Construction of Physical Map and QTL Analysis

SNP genotyping of parental lines CSR27, CSR11 and MI48 and the bulks of extreme tolerant and extreme susceptible RILs was done using the 50K chip (OsSNPnks). It was noticed that the sensitive parent MI48 (P_2_) used here was not the exact match with the MI48 used in the original cross to develop the mapping population, so if there was polymorphism between BT and BS and with monomorphic parental lines P_1_ and P_2_, then we considered the data on P_1_ (CSR11 and CSR27) as true result and assigned the alternate alleles to P_2_, as ignoring these loci may result in loss of important QTL regions. A QTL was called if homogeneous alleles were present in both tolerant and sensitive, either contributed by tolerant or susceptible parent. Sorting of polymorphic locus was done on Microsoft Excel sheet on the basis of chromosome number and then by their physical position in ascending order. Physical map of QTL regions was prepared in MapChart2.2, polymorphic homogeneous SNPs were color coded as red or green (Red background, tolerance allele was contributed by the sensitive parent; green background, tolerance allele was contributed by the tolerant parent).

## Supporting Information

S1 Fig Frequency distribution of lines for SSI for grain yield in CSR11/MI48 RILs. Parental lines and RILs derived from the cross between salt tolerant CSR11 (P_1_) and salt-sensitive MI48 (P_2_) were evaluated during 2009, 2010 and 2011 under moderate and high sodicity. The RILs showed significant variability for the nine salt tolerance parameters evaluated. All the parameters showed transgressive segregation and near normal distribution, suggesting involvement of multiple genes with quantitative inheritance. Tolerant CSR11 showed the least SSI in moderate (0.44) and high sodicity (0.75) as compared to the sensitive MI48 with SSI of 1.24 and 1.02, respectively across the three seasons.
(TIF)

S2 Fig Performance of the top 30 and bottom 30 lines for SSI for grain yield. The extreme top and bottom 30 RILs of CSR11/MI48 population were selected on the basis of their consistence SSI for grain yield undermoderate and high soidicity in three seasons (2009, 2010 and 2011). Red Bar, Top 30 RILs; Blue bar, Bottom 30 RILs
(TIF)

S1 Table Analysis of variance for yield and yield related traits under control (pH ~ 7.5), moderate sodic (pH ~ 9.5) and high sodic (pH ~ 9.9) soils in CSR11/MI48 RIL population evaluated for three years. (DOCX)

S2 Table Phenotypic data of all the RILs of CSR11/MI48 from three year evaluations for stress susceptibility index. Green background shows slected extreme tolerant RILs; Red background shows selected extreme susceptible RILs; RIL, recombinant Inbred Lines; SSIMS, stress susceptibility index at moderate stress; SSIHS, stress susceptibility index at high stress
(XLSX)

S3 Table Depiction of 5,821 polymorphic SNP loci in the pools of 10, 20, 30, 40 and 50 RILs of CSR11/MI48 with increasing number of heterogeneous loci in larger pools. (XLSX)

S4 Table Analyzed 6,068 polymorphic SNP loci in CSR11/MI48 RIL population. Green and red shaded homogeneous SNPs represent QTL regions with contribution of tolerant allele from the tolerant and sensitive parent, respectively, where as plain text with heterogeneous SNPs shows lack of association with the trait.
(XLSX)

S5 Table A summary of QTLs regions identified in the CSR11/MI48 RILs, their contributing parent for the tolerant allele, physical positions and size of the QTL intervals in base pairs identified by BSA using 50K SNP array. (DOCX)

S6 Table Analysis of 5,021 polymorphic SNP loci in CSR27/MI48RIL population. Green and red shaded homogeneous SNPs represent QTL regions with contribution of tolerant allele from the tolerant and sensitive parent, respectively, where as plain text with heterogeneous SNPs shows lack of association with the trait.
(XLSX)

S7 Table A summary of QTLs regions identified in the CSR27/MI48 RILs, their contributing parent for the tolerant allele, physical positions and size of the QTL intervals in base pairs identified by BSA using 50K SNP array. (DOCX)

S8 Table Recombination based scores for the QTLs identified in CSR27/MI48 and CSR11/MI48 derived mapping populations based on the genetic constitution of the flanking markers. (DOCX)
