# Supplementary figures and images for: Mapping QTLs for Salt Tolerance in Rice (Oryza sativa L.) by Bulked Segregant Analysis of Recombinant Inbred Lines Using 50K SNP Chip
Source: PLoS One. 2016 Apr 14;11(4):e0153610. doi: 10.1371/journal.pone.0153610 (PMC4831760; doi:10.1371/journal.pone.0153610)

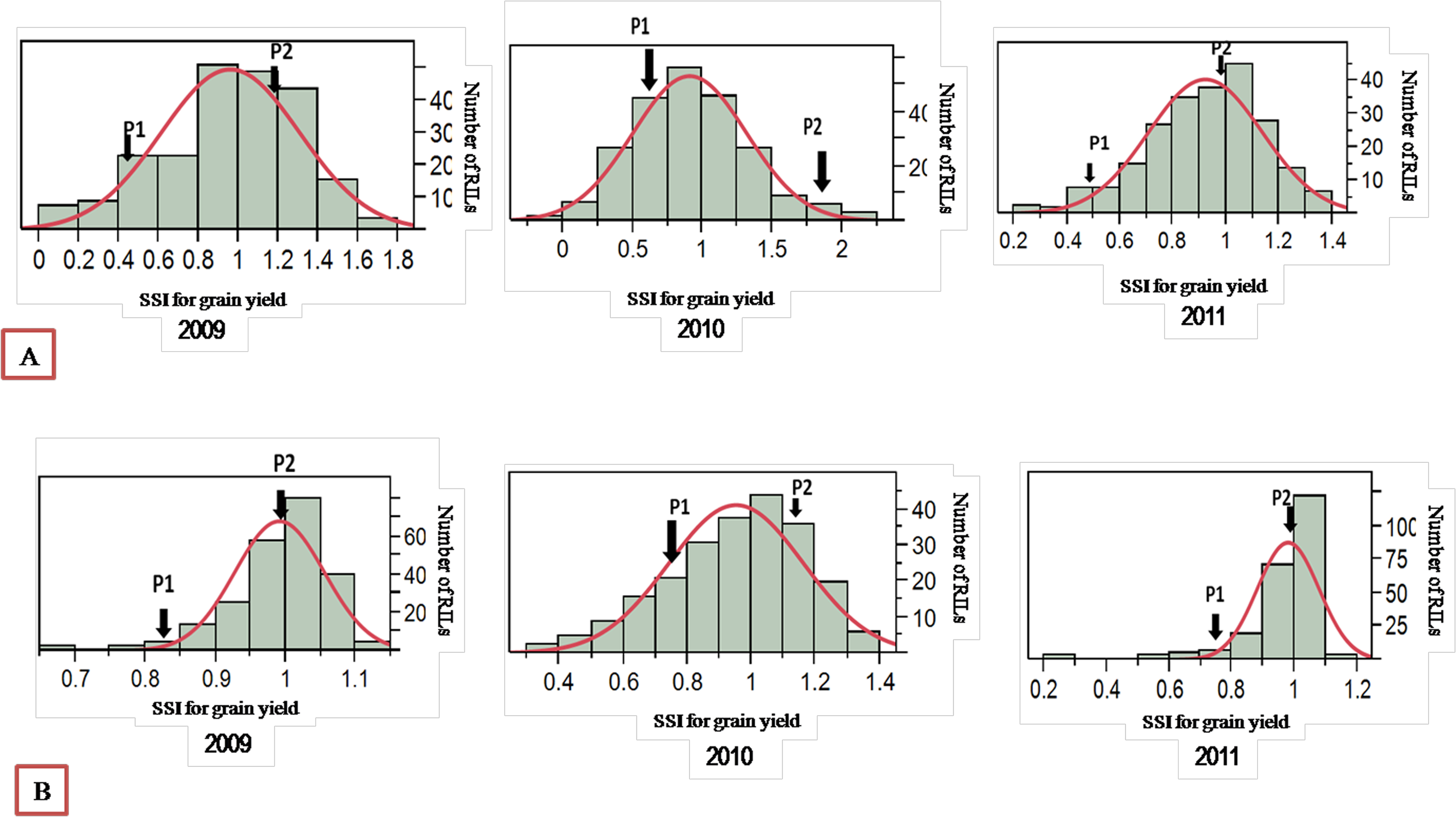

Supplement: S1 Fig — Parental lines and RILs derived from the cross between salt tolerant CSR11 (P1) and salt-sensitive MI48 (P2) were evaluated during 2009, 2010 and 2011 under moderate and high sodicity. The RILs showed significant variability for the nine salt tolerance parameters evaluated. All the parameters showed transgressive segregation and near normal distribution, suggesting involvement of multiple genes with quantitative inheritance. Tolerant CSR11 showed the least SSI in moderate (0.44) and high sodicity (0.75) as compared to the sensitive MI48 with SSI of 1.24 and 1.02, respectively across the three seasons. (TIF) [file pone.0153610.s001.tif]

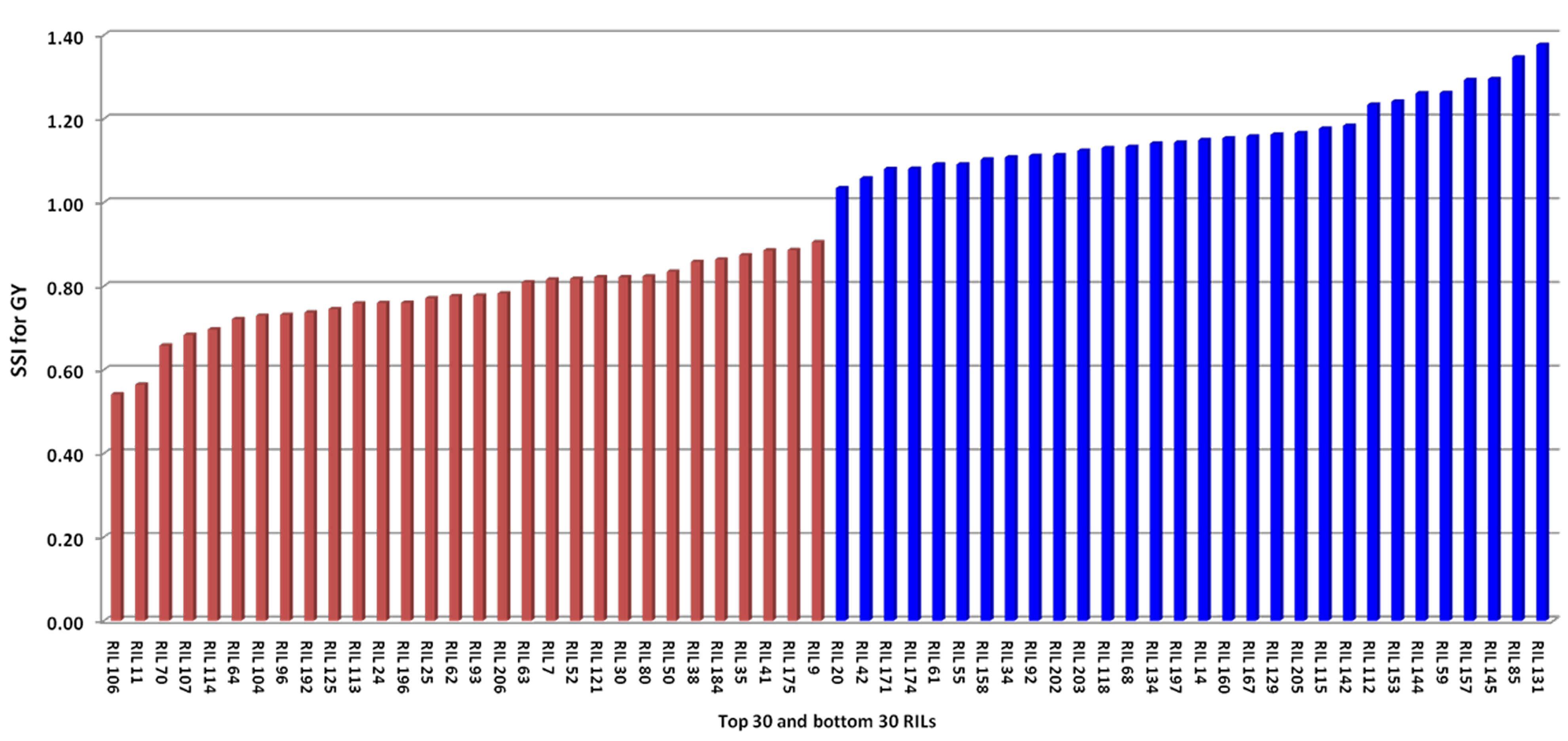

Supplement: S2 Fig — The extreme top and bottom 30 RILs of CSR11/MI48 population were selected on the basis of their consistence SSI for grain yield undermoderate and high soidicity in three seasons (2009, 2010 and 2011). Red Bar, Top 30 RILs; Blue bar, Bottom 30 RILs (TIF) [file pone.0153610.s002.tif]
