# Supplementary material for: Mapping QTLs for Salt Tolerance in Rice (Oryza sativa L.) by Bulked Segregant Analysis of Recombinant Inbred Lines Using 50K SNP Chip
Source: PLoS One. 2016 Apr 14;11(4):e0153610. doi: 10.1371/journal.pone.0153610 (PMC4831760; doi:10.1371/journal.pone.0153610)
Supplement: S1 Table — (DOCX) [file pone.0153610.s003.docx]

**Table S1.** Pooled analysis of variance for yield and related traits under normal (pH ~ 7.5), moderate sodic (pH ~ 9.5) and high sodic (pH ~ 9.9) soil in CSR11/MI48 RILs from three years data (2009, 2010, 2011)

| Source of variation | Stresses | df | Days to 50% flowering (DFF) | Plant height (PH) | Panicle length (PL) | Total tillers per plant (TT) | Productive tillers per plant (PT) | 1000 - grain weight (SW) | Grains per panicle (GPP) | Spikelet fertility (SF) | Grain yield per plant (GY) |
| --- | --- | --- | --- | --- | --- | --- | --- | --- | --- | --- | --- |
| Replications | N | 1 | 1.07 | 47.07 | 0.02 | 1.90 | 2.21 | 0.44 | 0.00 | 3.52 | 0.30 |
|  | MS |  | 52.44 | 23.99 | 1.01 | 3.10 | 2.76 | 0.41 | 307.43 | 73.77 | 2.00 |
|  | HS |  | 40.78 | 418.61** | 0.12 | 0.17 | 0.14 | 13.21 | 64.25 | 12.04 | 3.66 |
| Genotypes | N | 215 | 181.20** | 351.64** | 5.90** | 27.03** | 37.55** | 11.78** | 1388.28** | 436.82** | 165.15** |
|  | MS |  | 161.25** | 492.85** | 12.48** | 22.16** | 31.27** | 26.15** | 690.71** | 826.08** | 50.17** |
|  | HS |  | 139.65** | 327.51** | 21.38** | 7.12** | 8.88** | 35.81** | 434.41** | 394.55** | 8.63** |
| Seasons | N | 2 | 2.27** | 2.49 | 0.43 | 3.54** | 4.14** | 0.52** | 23.14** | 4.43** | 42.70** |
|  | MS |  | 15.48** | 120.7** | 2.12** | 1.12** | 2.76** | 2.18** | 49.42** | 48.70** | 0.35** |
|  | HS |  | 1.37 | 4.23** | 1.37** | 2.89** | 1.14 | 3.27** | 125.47** | 23.73** | 0.81** |
| Genotypes × Seasons | N | 430 | 0.16 | 1.94 | 0.22 | 0.01 | 0.08 | 0.03 | 0.52 | 0.27 | 0.11 |
|  | MS |  | 0.4733 | 1.0258 | 0.1516 | 0.0287 | 0.0303 | 0.1168 | 2.7737 | 0.8802 | 0.0612 |
|  | HS |  | 3.79 | 0.69 | 0.06 | 0.29 | 0.22 | 0.05 | 0.51 | 1.49 | 0.010 |
| Error | N | 766 | 52.57 | 122.47 | 2.19 | 4.71 | 4.26 | 3.49 | 340.08 | 52.04 | 29.10 |
|  | MS |  | 44.62 | 77.15 | 2.10 | 3.04 | 2.98 | 3.27 | 154.85 | 75.76 | 5.63 |
|  | HS |  | 39.88 | 33.68 | 2.23 | 1.72 | 1.48 | 3.41 | 99.78 | 98.84 | 1.17 |

N, Normal; MS, Moderate Stress; HS, High Stress; * and ** significant at 0.1 and 0.5 level respectively.
