# Supplementary material for: Mapping QTLs for Salt Tolerance in Rice (Oryza sativa L.) by Bulked Segregant Analysis of Recombinant Inbred Lines Using 50K SNP Chip
Source: PLoS One. 2016 Apr 14;11(4):e0153610. doi: 10.1371/journal.pone.0153610 (PMC4831760; doi:10.1371/journal.pone.0153610)
Supplement: S5 Table — (DOCX) [file pone.0153610.s007.docx]

| Chr. number | QTL Name | Physical position of homogeneous SNP  in Kb | Homogeneous SNP Gene ID | Gene product | Source of tolerant allele |
| --- | --- | --- | --- | --- | --- |
| 1 | qSSIGY1.1 | 32321638 | Os01g55530 | AP003256-AK101847 - NBS/LRR genes that are S-rich,divergent TIR, divergent NBS, expressed | CSR11 |
|  | qSSIGY1.2 | 34979206 | Os01g59930 | NADH-cytochrome b5 reductase, putative, expressed | CSR11 |
|  | qSSIGY1.3 | 39456907 | Os01g67370 | Expressed protein | CSR11 |
| 2 | qSSIGY2.1 | 9850734 | Os02g17190 | MYB family transcription factor | CSR11 |
|  | qSSIGY2.2 | 23107256 | Os02g38210 | Elongation factor Tu, putative, expressed | MI48 |
|  | qSSIGY2.3 | 34790405 | Os02g56750 | OsFBX65 - F-box domain containing protein, expressed | CSR11 |
| 3 | qSSIGY3.1 | 18724655 | Os03g32790 | expressed protein | CSR11 |
|  | qSSIGY3.2 | 19200676 | Os03g33650 | AGO7, putative, expressed | MI48 |
|  | qSSIGY3.3 | 26333713 | Os03g46610 | DEAD-box ATP-dependent RNA helicase, putative, expressed | MI48 |
|  | qSSIGY3.4 | 31508992 | Os03g55490 | Protein casein kinase II subunit alpha-2, putative, expressed | MI48 |
| 5 | qSSIGY5.1 | 23498993 | Os05g40150 | RGH2B, putative, expressed | CSR11 |
|  | qSSIGY5.2 | 26094905 | Os05g45040 | OsFBX170 - F-box domain containing protein, expressed | CSR11 |
|  | qSSIGY5.2 | 26097897 | Os05g45050 | Reticulon domain containing protein, putative, expressed | CSR11 |
| 6 | qSSIGY6.1 | 875250 | Os06g02530 | Expressed protein | CSR11 |
|  | qSSIGY6.2 | 12057391 | Os06g20870 | pentatricopeptide repeat protein PPR1106-17, putative, expressed | MI48 |
|  | qSSIGY6.3 | 19826152 | Os06g34040 | DJ-1 family protein, putative, expressed | MI48 |
|  | qSSIGY6.4 | 21954416 | Os06g37150 | L-ascorbate oxidase precursor, putative, expressed | CSR11 |
|  | qSSIGY6.5 | 29417258 | Os06g48620 | 4-amino-4-deoxychorismate synthase, putative, expressed | MI48 |
| 8 | qSSIGY8.1 | 3362109 | Os08g06110 | MYB family transcription factor, putative, expressed | MI48 |
| 9 | qSSIGY9.1 | 1279657 | Os09g02790 | zinc-binding protein, putative, expressed | MI48 |
| 12 | qSSIGY12.1 | 23122957 | Os12g37710 | PsbP, putative, expressed | MI48 |
