# Supplementary material for: Mapping QTLs for Salt Tolerance in Rice (Oryza sativa L.) by Bulked Segregant Analysis of Recombinant Inbred Lines Using 50K SNP Chip
Source: PLoS One. 2016 Apr 14;11(4):e0153610. doi: 10.1371/journal.pone.0153610 (PMC4831760; doi:10.1371/journal.pone.0153610)
Supplement: S7 Table — (DOCX) [file pone.0153610.s009.docx]

| **Chr. number** | **QTL Name** | **Physical position of homogeneous SNP in Kb** | **Homogeneous SNP Gene ID** | **Gene product** | **Source of tolerent allele** |
| --- | --- | --- | --- | --- | --- |
| 1 | qSSIGY1.1 | 30098494 | Os01g51770 | Outer mitochondrial membrane porin, putative, expressed | MI48 |
| 2 | qSSIGY2.1 | 22355864 | Os02g37020 | Expressed protein | MI48 |
|  | qSSIGY2.2 | 23667991 | Os02g39190 | Expressed protein | CSR27 |
|  | qSSIGY2.3 | 26780939 | Os02g44270 | Expressed protein | MI48 |
| 3 | qSSIGY3.1 | 7058058 | Os03g13100 | Ariadne-1, putative, expressed | CSR27 |
|  | qSSIGY3.2 | 7341998 | Os03g13614 | Transcription factor, putative, expressed | CSR27 |
|  | qSSIGY3.3 | 14277293 | Os03g25050 | Chaperonin, putative, expressed | MI48 |
|  | qSSIGY3.4 | 17474636 | Os03g30740 | Expressed protein | MI48 |
|  | qSSIGY3.4 | 17947278 | Os03g31550 | Aldehyde oxidase, putative, expressed | MI48 |
|  | qSSIGY3.5 | 23953725 | Os03g43020 | Prefoldin subunit, putative, expressed | MI48 |
|  | qSSIGY3.6 | 28577214 | Os03g50190 | Expressed protein | MI48 |
|  | qSSIGY3.7 | 30428413 | Os03g53140 | Protein transport protein Sec23B, putative, expressed | CSR11 |
|  | qSSIGY3.8 | 31508992 | Os03g55490 | Casein kinase II subunit alpha-2, putative, expressed | MI48 |
|  | qSSIGY3.9 | 35599824 | Os03g63074 | Ser/Thr protein phosphatase family protein, expressed | MI48 |
| 5 | qSSIGY5.1 | 2842030 | Os05g05740 | Proline synthetase co-transcribed bacterial homolog protein, putative, expressed | MI48 |
|  | qSSIGY5.2 | 5890481 | Os05g10730 | ABC transporter, ATP-binding protein, putative, expressed | MI48 |
|  | qSSIGY5.3 | 16848596 | Os05g28870 | Mitochondrial carrier protein, putative, expressed | CSR11 |
|  | qSSIGY5.4 | 23578540 | Os05g40270 | Expressed protein | MI48 |
|  | qSSIGY5.5 | 28139991 | Os05g49180 | Protein of unknown function DUF1296 domain containing protein, expressed | MI48 |
|  | qSSIGY5.6 | 29274051 | Os05g51180 | Hyaluronan/mRNA binding family domain containing protein, expressed | CSR11 |
| 6 | qSSIGY6.1 | 4394085 | Os06g08790 | ORC1 - Putative origin recognition complex subunit 1, expressed | MI48 |
|  | qSSIGY6.2 | 9268755 | Os06g16280 | Expressed protein | CSR11 |
|  | qSSIGY6.3 | 10758612 | Os06g18930 | Cadmium tolerance factor, putative, expressed | MI48 |
|  | qSSIGY6.4 | 28696344 | Os06g47340 | Glycosyltransferase family 43 protein, putative, expressed | MI48 |
|  | qSSIGY6.5 | 29417258 | Os06g48620 | 4-amino-4-deoxychorismate synthase, putative, expressed | MI48 |
| 8 | qSSIGY8.1 | 3132865 | Os08g05840 | DNA topoisomerase 1, putative, expressed | CSR27 |
|  | qSSIGY8.2 | 7940899 | Os08g13360 | Kelch repeat protein, putative, expressed | CSR27 |
|  | qSSIGY8.3 | 14794463 | Os08g24700 | Expressed protein | MI48 |
| 9 | qSSIGY9.1 | 11916388 | Os09g19920 | Metallo-beta-lactamase family protein, putative, expressed | MI48 |
|  | qSSIGY9.2 | 20070267 | Os09g33992 | Expressed protein | CSR27 |
| 11 | qSSIGY11.1 | 7519946 | Os11g13694 | AGAP007117-PA, putative, expressed | CSR27 |
| 12 | qSSIGY12.1 | 9359031 | Os12g16350 | Enoyl-CoA hydratase/isomerase family protein, putative, expressed | MI48 |
|  | qSSIGY12.2 | 23519826 | Os12g38380 | Tetratricopeptide repeat containing protein, putative, expressed | MI48 |
|  | qSSIGY12.3 | 24674134 | Os12g39980 | Kinesin motor domain containing protein, putative, expressed | MI48 |
