# Supplementary material for: Mapping QTLs for Salt Tolerance in Rice (Oryza sativa L.) by Bulked Segregant Analysis of Recombinant Inbred Lines Using 50K SNP Chip
Source: PLoS One. 2016 Apr 14;11(4):e0153610. doi: 10.1371/journal.pone.0153610 (PMC4831760; doi:10.1371/journal.pone.0153610)
Supplement: S8 Table — (DOCX) [file pone.0153610.s010.docx]

| **CSR 11/MI 48** | | | **CSR 27/MI 48** | | |
| --- | --- | --- | --- | --- | --- |
| **Chromosome** | **QTLs** | **Recombination Score** | **Chromosome** | **QTLs** | **Recombination Score** |
| 1 | 3 | 2 | 1 | 1 | 3 |
|  |  | 2 |  |  |  |
|  |  | 2 |  |  |  |
| 2 | 3 | 1.5 | 2 | 3 | 1.5 |
|  |  | 2.5 |  |  | 2 |
|  |  | 3 |  |  | 3 |
| 3 | 5 | 2 | 3 | 10 | 3 |
|  |  | 3 |  |  | 3.5 |
|  |  | 3 |  |  | 2.5 |
|  |  | 2 |  |  | 2 |
|  |  | 2.5 |  |  | 2 |
|  |  |  |  |  | 2.5 |
|  |  |  |  |  | 2 |
|  |  |  |  |  | 2 |
|  |  |  |  |  | 1.5 |
|  |  |  |  |  | 3 |
| 4 | None | - | 4 | None | - |
| 5 | 2 | 2 | 5 | 6 | 2 |
|  |  | 3 |  |  | 1.5 |
|  |  |  |  |  | 3 |
|  |  |  |  |  | 2.5 |
|  |  |  |  |  | 2.5 |
|  |  |  |  |  | 2.5 |
| 6 | 5 | 3 | 6 | 5 | 2.5 |
|  |  | 2.5 |  |  | 3 |
|  |  | 3.5 |  |  | 3 |
|  |  | 3.5 |  |  | 2.5 |
|  |  | 2 |  |  | 1.5 |
| 7 | None | - | 7 | None | - |
| 8 | 1 | 3 | 8 | 3 | 2.5 |
|  |  |  |  |  | 3 |
|  |  |  |  |  | 3 |
| 9 | 1 | 1.5 | 9 | 2 | 2 |
|  |  |  |  |  | 2.5 |
| 10 | None | - | 10 | None | - |
| 11 | None | - | 11 | 1 | 2 |
| 12 | 1 | 2 | 12 | 3 | 3 |
|  |  |  |  |  | 1.5 |
|  |  |  |  |  | 2.5 |
| **Total** | **21** |  | **Total** | **34** |  |
